# The polygenetically inherited metabolic syndrome of male WOKW rats is associated with enhanced autophagy in adipose tissue

**DOI:** 10.1186/1758-5996-5-23

**Published:** 2013-05-13

**Authors:** Joanna Kosacka, Karoline Koch, Martin Gericke, Marcin Nowicki, John T Heiker, Ingrid Klöting, Michael Stumvoll, Matthias Blüher, Nora Klöting

**Affiliations:** 1 Department of Medicine, Endocrinology and Diabetes, University Leipzig, Leipzig, Germany; 2 IFB AdiposityDiseases, Junior Research Group 2 “Animal models of obesity”, University Leipzig, Leipzig, Germany; 3 Institute of Anatomy, University Leipzig, Leipzig, Germany; 4 Department of Laboratory Animal Science, University of Greifswald, Greifswald, Germany; 5 IFB AdiposityDiseases, University Leipzig, Leipzig, Germany

**Keywords:** Autophagy, WOKW, Insulin resistance, Adipose tissue, Atg5, Atg7

## Abstract

**Background:**

Recent studies revealed that autophagy is up-regulated in obese individuals, as evidenced by increased expression of autophagy related genes. As argued elsewhere, it is possible that initially insulin resistance functions as an adaptive mechanism to increase autophagy in order to protect cells against death. We have shown that Wistar Ottawa Karlsburg W (RT1^u^) rats (WOKW) develop a metabolic syndrome with insulin resistance in adipose tissue, closely resembling the human disease. Therefore, the aim of this study was to characterize the autophagy phenotype in WOKW rats to clarify the interrelation between insulin resistance and autophagy in adipose tissue.

**Methods:**

Subcutaneous and epidydimal adipose tissue samples of 5-months-old WOKW and healthy LEW.1 W male rats were investigated and protein levels (Western blot and immunhistochemistry) of key autophagy genes, including Atg5, Atg7, LC3-II/LC3-I and apoptosis marker cleaved caspase-3 were analyzed.

**Results:**

WOKW rats displayed a significant increase of autophagy related proteins (Atg5, Atg7) in adipose tissue compared with LEW.1 W. This increase was predominantly found in epididymal adipose tissue. Furthermore, the LC3-II/LC3-I ratio as a marker of autophagosomes was significantly up-regulated in subcutaneous adipose tissue of WOKW rats. Cleaved caspase-3 was just slightly detectable in visceral adipose tissue and not detected in subcutaneous fat.

**Conclusion:**

Insulin resistance in adipose tissue of obese WOKW rats is associated with up-regulation of differing autophagy markers in visceral and subcutaneous fat depots. This fact not only qualifies the WOKW rat for further detailed analysis of genetic determinants of metabolic syndrome but also highlights its suitability for autophagy research.

## Introduction

Autophagy, or cellular self-digestion, is a catabolic process which targets cell constituents (damaged organelles, unfolded or misfolded proteins, intracellular pathogens) to lysosomes for degradation [1,2]. Under basal conditions, autophagy is involved in the degradation of long-lived proteins whereas the ubiquitinproteasome pathway, a different catabolic process, is responsible for the degradation of short-lived proteins [3,4]. The formation of the phagophore and the autophagosomes requires 18 distinct Atg proteins (AuTophaGyrelated) initially identified in yeast [5]. The process of autophagosome formation involves initiation, nucleation and elongation/enclosure [6]. The initiation step is controlled by the ULK1–Atg13–FIP200 complex and the nucleation step requires the Beclin-1–class III phosphatidylinositol 3-kinase (PI3K) complex. The two conjugation systems are divided in the elongation/enclosure step. The first is the conjugation of Atg12-Atg5 mediated by two ligases Atg7 and Atg10 and the second involves cleavage of microtubule-associated protein 1 light chain 3 (LC3 or Atg8) by Atg4 releasing the soluble form LC3-I, which is then conjugated to phosphatidylethanolamine (PE) via participation of Atg7 and Atg3. This lipid conjugation forms the autophagic double membrane-associated LC3-II protein allowing the closure of autophagic vacuole [6]. LC3-II is used as a marker of autophagosomes [7]. The main inhibitor of autophagy is the serine/threonine kinase mammalian target of rapamycin (mTOR), a component of the mTORC1 complex [6].

As a survival mechanism, dysregulated autophagy has been linked to many human pathophysiologies, such as cancer, myopathies, neurodegeneration, heart and liver diseases [8]. Interestingly, recent studies revealed that autophagy is up-regulated in obese individuals, as evidenced by increased expression of autophagy gene ATG5, LC3A, and LC3B as well as elevated autophagic flux in omental and subcutaneous adipose tissue [9]. Moreover, Codogno and Meijer argued that initially obesity-induced insulin resistance functions as an adaptive mechanism to increase autophagy in order to protect cells against death [10]. They further discussed that defective autophagy may also underlie impaired insulin sensitivity in obesity and that up-regulating autophagy can combat insulin resistance [10].

Wistar Ottawa Karlsburg W (RT1^u^) rats (WOKW) develop a complete metabolic syndrome with hypertension, impaired glucose tolerance, hyperinsulinaemia, dyslipidemia as well as insulin resistance in adipose tissue, closely resembling the human disease [11-16]. Crossing studies confirmed that the metabolic syndrome of the WOKW rat is under polygenic control and occurs in a gender-dependent manner [16].

This relationship between increased autophagy and obesity poses the question on a possible role of autophagy in the state of obesity-induced insulin resistance in the adipose tissue of WOKW rats. The aim of this study was to characterize the autophagy phenotype of adipose tissue in WOKW rats and clarify if there is a link between insulin resistance and autophagy.

## Material and methods

### Animals

In 2011, breeding pairs of LEW.1 W (F105 + 2) and WOKW (F102 + 2) rats from the Laboratory of Animal Science, University of Greifswald, were obtained and bred in our own animal facility under standardized environmental conditions. Nine male rats of both strains were used for these experiments. Animals were kept in groups of four in Macrolon cages (Size 3, Ehret, Emmendingen, Germany) under strict hygienic conditions. They had free access to food and water and were maintained at a 12-h light and dark cycle (5 AM/5 PM). All experiments were conform to the Guide for the Care and Use of Laboratory Animals published by the US National Institutes of Health (NIH Publication No. 85–23, revised 1996) and were approved by the local authorities of the state of Saxony, Germany as recommended by the responsible local animal ethics review board (T58/12).

### Phenotypic characterization

Two weeks before killing, all phenotypic characterizations were carried out. Briefly, blood samples were obtained from animals by puncturing the ophthalmic venous plexus after 12 hours of fasting for determination of blood glucose, serum triglycerides, total cholesterol, and serum insulin. Blood glucose was determined using a glucose analyser (Freestyle Mini, Abbott, Wiesbaden, Germany). Serum triglycerides and total cholesterol were analysed using an automatic analyzer (Roche Cobas Mira Plus, Roche, Switzerland). Serum insulin was determined using enzyme immunoassay kit (Rat Insulin ELISA, Mercodida AB, Uppsala, Sweden). For calculating the body mass index (BMI), the body length of animals was measured. At the end of observation period left and right inguinal adipose tissue pads were removed and weighed. The sum of fat pads to body weight multiplied by 100 gave the adiposity index.

### Western blot

Animals were sacrificed and dorsal subcutaneous fat pads and epididymal fat pads were removed. Tissues were lysed by ultrasonication in 60 mMTris-HCl, pH 6.8, containing 2% sodium dodecyl sulfate (SDS) and 10% sucrose. Tissue lysates were diluted 1:1 in sample buffer (250 mMTris-HCl, pH 6.8, containing 4% SDS, 10% glycerol, and 2% b-mercaptoethanol) and denatured at 95°C for 5 min. Protein concentration was assessed with the BCA protein assay (Pierbo Science, Bonn, Germany). Proteins (30 μg per lane) were separated by electrophoresis on 12.5% or 15% SDS-polyacrylamide gels and transferred to nitrocellulose membranes by electroblotting. Nonspecific binding sites were blocked with 5% dry milk for 45 min. The blots were incubated with different antibodies at 4°C overnight: mouse anti-LC3 (1:500; clone 2G6; Nanotools, Munich, Germany), polyclonal rabbit anti-cleaved caspase-3, Atg5 (D1G9) and Atg7 (all 1:1 000; Cell Signaling Technology, Danvers, USA). Proteins were detected by incubating with HRP conjugated secondary antibodies (1:4 000 dilution; Dianova) at RT for 2 h and chemiluminescence kit (Amersham, Pharmacia, Freiburg, Germany). Integrated optical densities of the immunoreactive protein bands were measured with Gel Analyzer software (Media Cyberneties, Silver Spring, MD). Equal protein loading was verified using mouse anti-D-glyceraldehyde-3-phosphate dehydrogenase antibody (GAPDH, Research Diagnostics, Flanders, The Netherlands; 1:3 000). The extract of Jurkat cells with cytochrome c-induced apoptosis (Cell Signaling Technology) was used as positive control for the detection of cleaved caspase-3.

### Immunofluorescence staining for Atg5, Atg7, LC3 and cleaved caspase-3

The subcutaneous and epididymal adipose tissues were fixed in buffered 10% Zinc-formalin. The 20-μm-thick frozen sections from the epididymal and subcutaneous fat padswere mounted on gelatinized glass slides. After buffer rinse, sections were incubated with mouse anti-LC3 (1:250; clone 2G6; Nanotools,), Atg5 (D1G9), Atg7 (all 1:500; Cell Signaling Technology) and polyclonal rabbit anti-cleaved caspase-3 (1:500) at 4°C overnight. After buffer rinse, FITC-conjugated goat anti-rabbit IgG was diluted 1:2000, and sections were incubated at room temperature for another 2 h. Sections were mounted with DakoGlycergel (DakoCytomation) containing 10 μg/ml DAPI (Serva, Heidelberg, Germany) for nuclear staining and 25 μg/ml DABCO (Sigma) to prevent photobleaching. By replacement of the primary antisera with normal mouse IgG, rabbit serum or PBS, respectively, no specific immunoreaction occurred.

### Data analysis and statistics

Data are given as means ± SD unless stated otherwise. Data sets were analyzed using a two-tailed unpaired *t* test, or differences were assessed by one-way ANOVA using the Statistical Package for Social Science, version 18.0 (SPSS, Chicago, IL). *P* values <0.05 were considered significant.

## Results

### Phenotype of WOKW rats

The phenotypic characterization of the 5-month-old WOKW and LEW.1 W rats studied is summarized in Table 1. WOKW rats have a significantly higher body weight, BMI, fasting serum insulin as well as serum triglyceride levels compared to the healthy LEW.1 W rats. In contrast, HbA1c levels and blood glucose did not differ between both strains. The adiposity index was significantly increased in WOKW compared to LEW.1 W rats.

**Table 1:** Phenotype of LEW.1 W and WOKW rats at an age of 5 months (means ± SD)

**Traits**	**LEW.1 W (n** = **9)**	**p-value**	**WOKW (n** = **9)**
Body weight (g)	486 ± 15	< 0.001	712 ± 21
BMI (kg/cm^2^)	0.68 ± 0.15	0.017	0.85 ± 0.12
Blood glucose (mmol/l)	5.8 ± 0.5	NS	6.5 ± 0.9
HbA1c (%)	3.70 ± 0.11	NS	3.72 ± 0.12
Serum insulin concentration (ng/ml)	2.1 ± 0.8	<0.001	10.1 ± 3.1
Triglycerides (mmol/l)	1.5 ± 0.5	<0.001	3.9 ± 0.5
Total cholesterol (mmol/l)	3.2 ±0.6	NS	2.8 ± 0.4
Adiposity index	1.1 ± 0.6	<0.001	3.5 ±0.6

### Enhanced autophagy in epidydimal adipose tissue of WOKW rats

**Figure 1:**
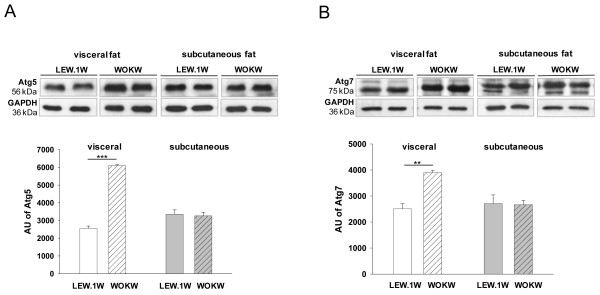
**Expression of autophagy markers Atg5 and Atg7 in visceral und subcutaneous fat of WOKW and LEW.1 W rats.** Representative Western blots and corresponding densitometrical analyses (Figure 1**A**, **B**) of Atg5 and Atg7 in visceral and subcutaneous fat of WOKW rats compared to the LEW.1 W control animals. The significant increased expression of Atg5 and Atg7 in visceral fat WOKW rats vs. LEW.1 W control animals. Data from n = 6 are presented as mean ± SEM * p ≤ 0.05, ** p ≤ 0.01, *** p ≤ 0.001, according to the one-way analysis of variance together with the Newman-Keuls test. GAPDH was used as loading control. AU – arbitrary units.

**Figure 2:**
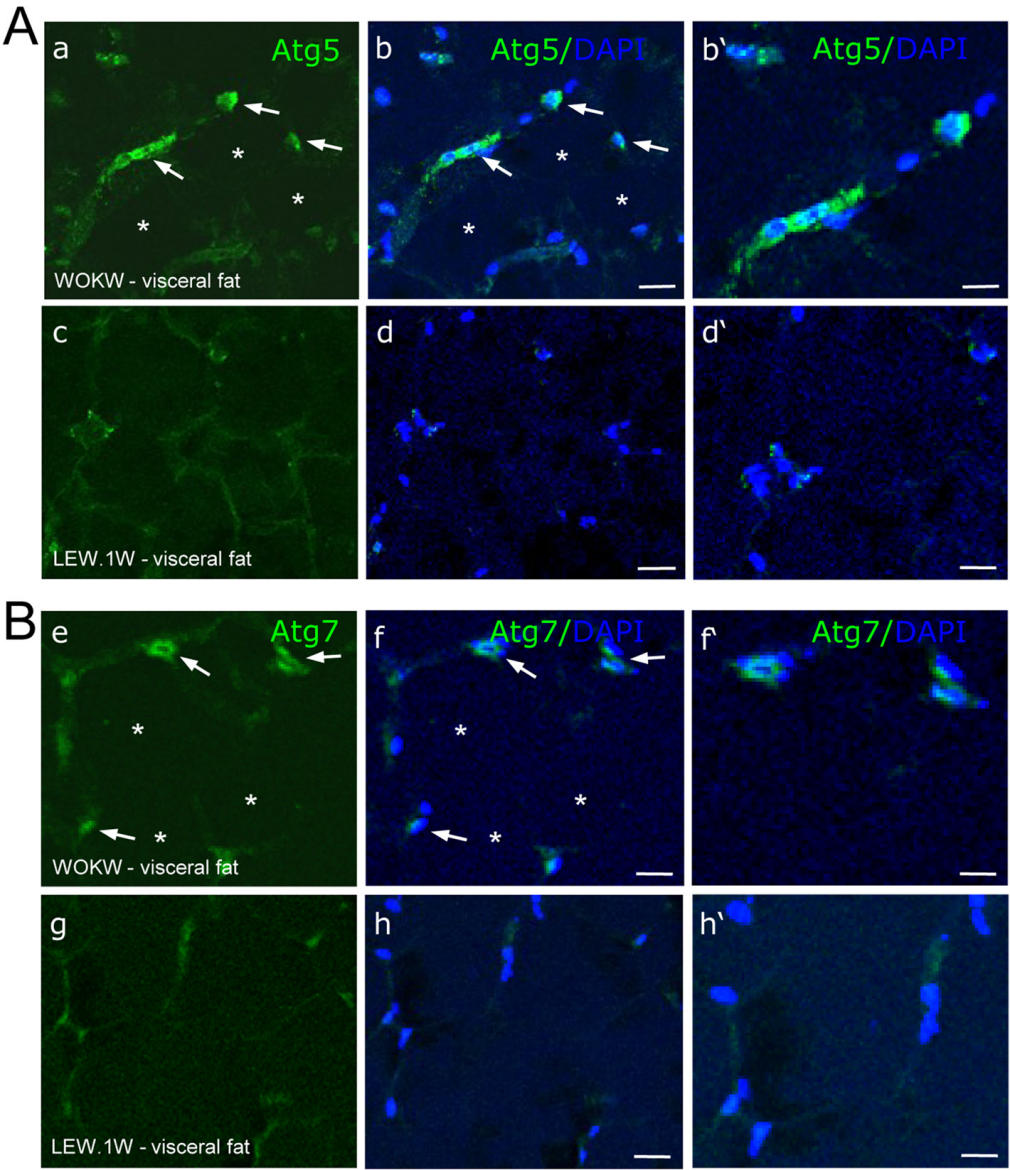
**Detection of Atg5 and Atg7 expression in visceral fat of WOKW and LEW.1 W rats by immunofluorescence.** A significantly higher immunofluorescence intensity of Atg5 and Atg7 was found in visceral fat of WOKW rats compared with LEW.1 W control animals (Figure 2**A**, **B**). Both proteins could only be detected in stromal cells of the adipose tissue (arrow), rather in adipocytes directly (star; Figure 2**B** and f). Scale bars: 15 μm (a - h); 30 μm (b’- h’).

We first assessed the protein level of key autophagy related genes, Atg5 and Atg7, in visceral and subcutaneous adipose tissue. As shown in Figure 1A and B, visceral adipose tissue of WOKW rats displays a significant increase in Atg5 and Atg7 protein expression compared to healthy lean LEW.1 W. Atg5 was 2.3-fold increased and Atg7 displayed a 2-fold up-regulation. The expression levels of Atg5 and Atg7 in subcutaneous adipose tissue were comparable between both strains. Immunofluorescence analysis confirmed the western blot results (Figure 2A-B).

### LC3-II/LC3-I ratio is up-regulated in SC fat tissue of WOKW rats while cleaved caspase-3 is not altered

**Figure 3:**
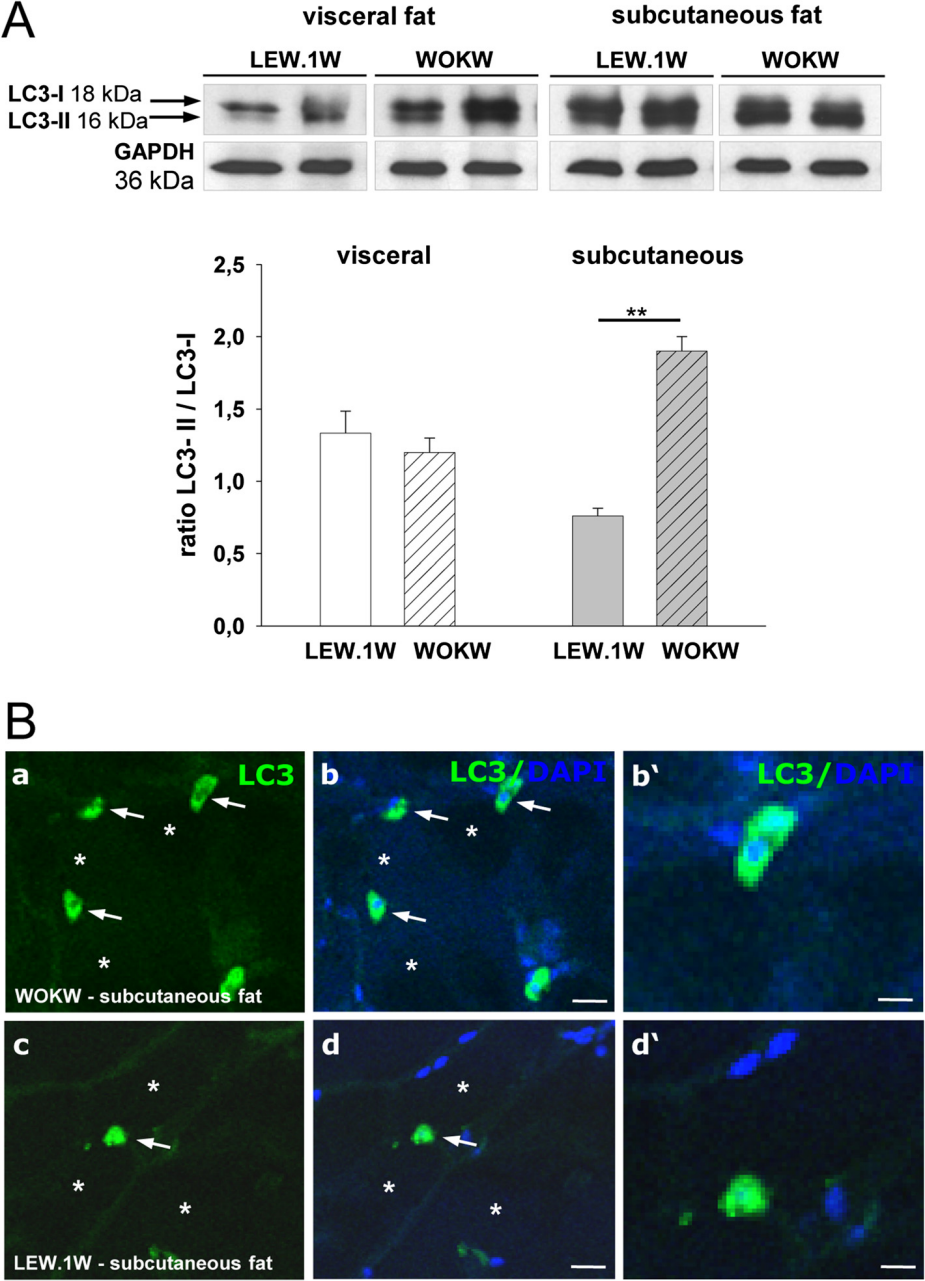
**Expression of LC3-I and LC3-II proteins in visceral und subcutaneous fat WOKW and LEW.1 W rats.** The over expression of the LC3-II (membrane bound) protein and a significantly higher LC3-II / LC3-I ratio in subcutaneous fat of WOKW rats vs. LEW.1 W control animals. Representative Western blots and corresponding densitometrical analyses (Figure 3**A**). The presence and location of the LC3 protein in subcutaneous fat of WOKW and LEW.1 W rats is additionally shown by immunostaining. As shown for Atg5 and Atg7, adipocytes were predominately negative for LC3 (star). Strong LC3 staining was found in stroma cells resembling macrophages (arrows; Figure 3**B**). Scale bars: 15 μm (a-d); 50 μm (b’-d’) (Figure 3**B**). Data from n = 6 are presented as mean ± SEM * p ≤ 0.05, ** p ≤ 0.01, *** p ≤ 0.001, according to the one-way analysis of variance together with the Newman-Keuls test. GAPDH was used as loading control.

**Figure 4:**
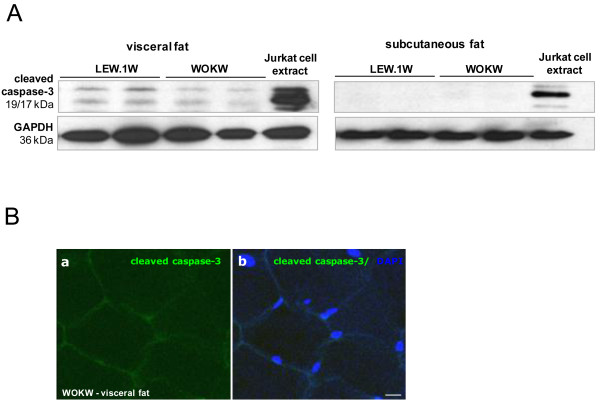
**Expression of apoptosis marker cleaved caspase-3.** The cleaved caspase-3 expression is slightly detectable by Western blot and immunofluorescence staining of visceral and subcutaneous fat of WOKW and LEW.1 W rats (Figure 4**A**, **B**). Bar represents 15 μm. The extract of Jurkat cells with cytochrome *c*-induced apoptosis was used in Western blot as the positive control for occurrence of cleaved caspase-3.

The analysis of the LC3-II/LC3-I ratio showed an up-regulation of autophagosomes in subcutaneous adipose tissue of WOKW rats (Figure 3A). Immunohistofluorescence analysis confirmed the western blot results (Figure 3B). Here, we detected a 2-fold increase in WOKW rats compared to control animals. In addition, cleaved caspase-3 as an apoptosis marker was just barely detectable in visceral adipose tissue and there was no signal of cleaved caspase-3 detected in subcutaneous fat depot (Figure 4A-B).

## Discussion

Recent studies revealed that autophagy is up-regulated in human obesity [9] and it has been shown that autophagy is more pronounced in omental than in subcutaneous adipose tissue [17]. In this study we demonstrate that insulin resistant adipose tissue of WOKW rats display enhanced autophagy with increased expression of autophagy genes. Markers of autophagy were different in omental than in subcutaneous adipose tissue. We found autophagy related genes more evidently up regulated in omental fat tissue of WOKW rates compared with LEW.1 W rats. In accordance with results from studies in human obesity, we found an increase of Atg5 and Atg7 expression in visceral adipose tissue of WOKW rats, suggesting parallel alterations in the endocrine function of adipose tissue both in human and in WOKW metabolic syndrome [9]. Among the many autophagy-related genes, Atg7, which encodes an ubiquitin-activating enzyme (E1)-like enzyme, is pivotal for autophagosome formation [18] and responsible for both Atg12-Atg5 conjugation and LC3 conversion. In livers of *ob*/*ob* mice it was shown that Atg7 is dramatically down-regulated suggesting that reconstitution of Atg7 expression would likely be an effective way to reestablish autophagy, at least in part, in liver [19]. In addition, blocking autophagy using small interfering RNA targeted to ATG7 in human Simpson-Golabi-Behmel syndrome adipocytes resulted in up-regulation of inflammatory marker [19]. These data indicates that autophagy may function to dampen inflammatory gene expression and thereby limit excessive inflammation in adipose tissue during obesity. Further studies are needed, to determine to what extend inflammatory markers in WOKW rats are regulated by increased autophagy in adipose tissues. Microtubule-associated protein 1 light chain 3 (LC3), a homologue of yeast Atg8 (Aut7/Apg8), localizes to autophagosomal membranes after post-translational modifications. LC3-I is cytosolic, whereas LC3-II is membrane bound [20]. The C-terminal fragment of LC3 is cleaved immediately following synthesis to yield a cytosolic form called LC3-I. A subpopulation of LC3-I is further converted to an autophagosome-associating form, LC3-II [20,21]. Accordingly, the amount of LC3-II correlates well with the number of autophagosomes [20]. This characteristic conversion of LC3 can be used to monitor autophagic activity [21]. Interestingly, autophagosomes measured as LC3-II/LC3-I ratio were significantly up-regulated only in subcutaneous adipose tissue of WOKW rats compared with healthy LEW.1 W rats. This is consistent with a human study from Jansen et al., showing that levels of the autophagy marker LC3 were elevated in subcutaneous adipose tissue of obese vs. lean human subjects and positively correlated to both systemic insulin resistance and morphological characteristics of adipose tissue inflammation [22]. It has been shown by Kadowaki and co-workers that LC3 ratio represents a reliable index of autophagy formation and is much more sensitive and proportional to changes in the proteolytic rates than the ratio from the total homogenate [23]. The macroautophagic degradation involves multiple steps and can occur by different signal transduction pathways. The formation of the isolation membrane of the autophagosome is the earliest event of macroautophagy, which is dependent of the Atg5/Atg7 protein expression. The interaction of Atg complex with LC3 protein and its lipidation to membrane associated LC3-II form occurs in the next steps [20,21]. The significantly up-regulated expression of Atg5/Atg7 proteins in visceral fat tissue of WOKW rats can indicate on the induction of autophagy, before the conversation of LC3 protein is detectable.

By contrast, in the subcutaneous adipose tissue, a higher LC3-II/LC3-I ratio without any regulation of Atg proteins expressions was detected. However, the presence of LC3 protein was only found in stroma cells resembling macrophages. The Atg5/Atg7-independent autophagic pathway with the LC3-positive and LC3-negative autophagosomes was discovered in mouse fibroblast lacking Atg5/Atg7 [24]. Moreover, LC3-associated phagocytosis (LAP) has been recently identified as a phenomenon distinct from classical autophagy [25]. The LC3-associated phagocytosis has been shown to be required for the efficient clearance of dead cells [26]. In the light of these findings, the occurrence of LC3-associated phagocytosis in subcutaneous adipose tissue of WOKW rats cannot be excluded and may be associated with the degradation of phagocytosed cellular corpses.

Interestingly, we found no differences in the amount of cleaved caspase-3 between WOKW and LEW.1 W rats. As an apoptosis marker, cleaved caspase-3 was barely observed in visceral adipose tissue and not detected in subcutaneous fat depot. In contrast, Alkhouri and co-workers showed that both mitochondrial- and death receptor-mediated caspase activation and adipocyte apoptosis were increased in the adipose tissue of obese humans and diet-induced obese mice [27]. Based on the obviously low levels of cleaved caspase-3 in adipose tissues of WOKW rats, it could be argued that up-regulation of autophagy protects from capase-3-mediated apoptosis.

## Conclusion

Polygenetically inherited metabolic syndrome of WOKW rats is associated with enhanced autophagy in insulin resistant adipose tissue. Furthermore, the increasing autophagic activity seems to protect from caspase-3 mediated apoptosis. These findings further implicate that the WOKW rat is a useful model for the human endocrine status in obesity, the metabolic syndrome and also highlights its suitability for autophagy research.

## Competing interests

The authors declare that they have no competing interests.

## Authors’ contributions

NK, IK and JK designed and planned the study. JK, KK and MN carried out the experimental work, biochemical and statistical analysis. NK, MS and JH performed interpretation and discussion of results. NK, MB and MG drafted and revised the manuscript. All authors read and approved the final manuscript.
